# Characterization of excitatory and inhibitory neuron activation in the mouse medial prefrontal cortex following palatable food ingestion and food driven exploratory behavior

**DOI:** 10.3389/fnana.2014.00060

**Published:** 2014-07-01

**Authors:** Ronald P. A. Gaykema, Xuan-Mai T. Nguyen, Jessica M. Boehret, Philip S. Lambeth, Jonathan Joy-Gaba, Daniel M. Warthen, Michael M. Scott

**Affiliations:** Department of Pharmacology, School of Medicine, University of Virginia, CharlottesvilleVA, USA

**Keywords:** vasoactive intestinal peptide, parvalbumin, somatostatin, palatable food seeking and ingestion, prefrontal cortex, prelimbic, infralimbic

## Abstract

The medial prefrontal cortex (mPFC) is implicated in aspects of executive function, that include the modulation of attentional and memory processes involved in goal selection. Food-seeking behavior has been shown to involve activation of the mPFC, both during the execution of strategies designed to obtain food and during the consumption of food itself. As these behaviors likely require differential engagement of the prefrontal cortex, we hypothesized that the pattern of neuronal activation would also be behavior dependent. In this study we describe, for the first time, the expression of Fos in different layers and cell types of the infralimbic/dorsal peduncular and prelimbic/anterior cingulate subdivisions of mouse mPFC following both the consumption of palatable food and following exploratory activity of the animal directed at obtaining food reward. While both manipulations led to increases of Fos expression in principal excitatory neurons relative to control, food-directed exploratory activity produced a significantly greater increase in Fos expression than observed in the food intake condition. Consequently, we hypothesized that mPFC interneuron activation would also be differentially engaged by these manipulations. Interestingly, Fos expression patterns differed substantially between treatments and interneuron subtype, illustrating how the differential engagement of subsets of mPFC interneurons depends on the behavioral state. In our experiments, both vasoactive intestinal peptide- and parvalbumin-expressing neurons showed enhanced Fos expression only during the food-dependent exploratory task and not during food intake. Conversely, elevations in arcuate and paraventricular hypothalamic fos expression were only observed following food intake and not following food driven exploration. Our data suggest that select activation of these cell types may be required to support high cognitive demand states such as observed during exploration while being dispensable during the ingestion of freely available food.

## INTRODUCTION

Through the receipt of information on taste and palatability from primary gustatory insular and secondary orbitofrontal taste cortices (Ongur and Price, 2000; Carleton et al., 2010) along with integration of information from other brain areas such as the amygdala and midline thalamus, the medial prefrontal cortex (mPFC; Uylings et al., 2003; Kesner and Churchwell, 2011) modulates food seeking behavior through excitatory projections to the striatum and in particular to the nucleus accumbens (Kelley, 2004). Consequently, an alteration in the function of this brain area would be expected to result in aberrant food intake and the development of feeding related pathologies. Indeed, functional magnetic resonance imaging (fMRI) of people exhibiting diseases of disordered feeding such as bulimia often show activity changes in prefrontal cortex such as in Brodmann’s area 32 (Marsh et al., 2009; Lock et al., 2011). Interestingly, anorexics and obese individuals show opposite changes in PFC activity as measured by fMRI in a taste reward task when compared to controls (Frank et al., 2012), suggesting that altered PFC function may be driving the observed pathological changes in feeding behavior. In the rodent, the importance of a homologous region of the prefrontal cortex [Uylings et al., 2003; Kesner and Churchwell, 2011; including the prelimbic/anterior cingulate (PL/AC) and infralimbic/dorsal peduncular (IL/DP) cortex] in driving food intake is highlighted by numerous studies, including those demonstrating the role of neuropeptides acting at prefrontal cortical μ-opioid receptors to modulate feeding (Mena et al., 2011; Blasio et al., 2013). Lesion studies, meanwhile, have shown the importance of the mPFC in cued feeding and in driving operant responding for palatable food (Petrovich et al., 2007a,b; Moscarello et al., 2010).

Interestingly, while the relevance of the prefrontal cortex in driving a variety of behaviors relevant to the pursuit of food reward has been well described, little is known about possible differential engagement of specific neuronal populations in the mPFC during exploratory palatable food seeking or ingestive behavior. This stands in stark contrast to areas of the hypothalamus that have been extensively studied, in particular the paraventricular (PVN), lateral hypothalamic and arcuate (Arc) nuclei, resulting in a detailed understanding of how this subcortical neuronal circuitry drives feeding behavior (as reviewed by Sohn et al., 2013). For example, proopiomelanocortin (POMC) and agouti-related peptide (AGRP) neuronal populations in the Arc nucleus have been described to differentially sense hormonal signals such as leptin (Williams et al., 2010; Baver et al., 2014) and ghrelin (Betley et al., 2013) to drive or inhibit feeding. PVN hypothalamic neurons have subsequently been shown to act as a convergence point between AGRP (Atasoy et al., 2012) and POMC (Balthasar et al., 2005) signaling to regulate food intake through projections to nucleus of the solitary tract hindbrain neurons. Lateral hypothalamic neurons, meanwhile, participate in the sensing of nutrient content through engagement of melanin-concentrating hormone peptide expressing neurons (Domingos et al., 2013) and in the determination of taste palatability (Li et al., 2013), possibly as a result of activation of orexin peptide expressing neurons.

One important question to be addressed regarding the description of neurons of the mPFC activated during reward driven exploration and reward consumption is whether specific types of γ-aminobutyric acid (GABA)-producing inhibitory neurons, involved in shaping excitatory output (Constantinidis et al., 2002), are selectively modulated in a manner dependent upon behavioral state. While excitatory pyramidal neurons would likely increase their activity to support greater mPFC output, it is difficult to predict how a heterogeneous population of GABAergic interneurons would react. For example, while parvalbumin (PV)-expressing basket type interneurons are tightly coupled to excitatory pyramidal cells, synchronizing their output (Somogyi et al., 1983; Cardin et al., 2009), many other classes of inhibitory interneurons (DeFelipe et al., 2013) contribute to the regulation of rhythmic pyramidal cell activity and shifts in the excitation-inhibition balance (Vogels and Abbott, 2009).For example, vasoactive intestinal peptide (VIP)-containing interneurons mainly exist as radially projecting bipolar neurons (and as small numbers of basket cells), which extend processes between cortical layers to innervate other groups of inhibitory interneurons (David et al., 2007; Pi et al., 2013). Consequently, these VIP interneurons have been suggested to promote cortical column output via a disinhibitory mechanism in the mPFC (Pi et al., 2013). The peptide somatostatin (SOM), meanwhile, is often expressed in Martinotti cells, which target pyramidal cell dendrites, and which are inhibited during specific aspects of reward-seeking behavior (Kvitsiani et al., 2013), while also showing consistent activation following exposure to a variety of stimuli (Fanselow et al., 2008). Thus, activation of SOM neurons is thought to be part of a mechanism that broadly reduces mPFC neuronal output.

The principal goal of our current work is to characterize the activation of specific populations of mPFC neurons, both glutamatergic and GABAergic, following food driven exploration and following food ingestion. We hypothesized that these behaviors would differentially engage subsets of mPFC neurons, based on prior data that foraging behavior produces a more complex pattern of mPFC activity than the simple act of food ingestion (Caracheo et al., 2013). In a test of our hypothesis, we investigated whether the molecularly distinct interneuron subtypes demarcated by the expression of either PV, SOM or VIP [peptides expressed by chemically distinct and non-overlapping populations that together represent the greater majority, up to 84%, of all cortical interneurons (Xu et al., 2010; Rudy et al., 2011; Pfeffer et al., 2013)], along with excitatory pyramidal neurons are differentially activated in defined regions of the mPFC following either food reward-driven exploratory behavior or consumption.

To assess neuronal activation, we have employed a well established method of immunohistochemical detection of Fos, the protein product of the immediate early gene c-*fos* (reviewed by Herrera and Robertson, 1996). Using this technique, prior work has shown how diverse stimuli differ in their ability to activate neurons of the mPFC, implying functional differences in terms of the neuronal circuitry engaged. For example, certain drugs of abuse such as nicotine, alcohol, amphetamines, and cocaine increase Fos expression in the mPFC (Schroeder et al., 2001; Miller and Marshall, 2004; Morshedi and Meredith, 2007; Leriche et al., 2008), although the cell types activated appear to differ substantially. Consequently, we performed an analysis of Fos expression in excitatory principal and inhibitory PV, SOM, and VIP-containing cell types within subdivisions of the mouse mPFC and report the first detailed characterization of immediate early gene expression in this brain area following food intake and food driven exploratory behavior.

## MATERIALS AND METHODS

### ANIMALS

Male mice C57BL6/J between 6 and 8 weeks old were purchased from The Jackson Laboratory and housed in groups of 4–5 upon arrival for at least a week. The mice were housed under standard climate-controlled conditions on a 12 h light–dark cycle (lights on at 9:00 AM) with standard rodent chow (Harlan Teklad 7012, 3.10 kcal/g) and water available *ad libitum*. Prior to experimentation the animals were separated and subsequently individually housed for at least another week. Animal use was in accordance with guidelines approved by the University of Virginia Animal Care and Use Committee.

### FOOD INTAKE ASSAY VALIDATION

Prior to Fos analysis, we established conditions that would reliably produce intake of palatable food, but not regular chow, by offering the food shortly after the onset of the light period, when the mice are generally sated after nocturnal feeding and show negligible metabolic drive to feed. The feeding assay was validated using a group of 12 B6 male mice that were offered at ZT = 0.5–1.5 h a pellet of either standard rodent chow (*n* = 6) or Surwit diet with high fat and sugar content (referred to as “high fat diet” or HFD; Research Diets Inc., D12331, 5.56 kcal/g, *n* = 6) in an open polystyrene dish bottom. The food pellets were removed 30 min later. The pellets were weighed before and after the feeding period to determine the amount consumed. To reduce the effect of novelty of the food on feeding behavior, the mice in the HFD group were offered a small piece of HFD (∼0.1 g) the afternoon prior to the experiment.

### FOOD INTAKE AND FOOD DRIVEN EXPLORATORY ACTIVITY ASSAY FOR BRAIN FOS ANALYSIS

After a full week of acclimatization to individual housing, a cohort of 15 B6 male mice were assigned to one of the three experimental groups: (a) the control group, in which mice were offered an empty, small polystyrene dish, (b) the HFD-fed group, in which mice were offered a pellet of HFD placed in an open polystyrene dish, and (c) the food-driven exploratory group, in which mice were offered a pellet of HFD locked inside polystyrene dish tightly shut with a perforated lid, enabling the mice to smell and pursue the food, but preventing them from consuming it. In order to get the mice acquainted and familiarized with the novel HFD, a small piece (∼0.1 g) was offered in the afternoon prior to the testing day. Mice were also offered the same type of dishes as were used during the experiment for familiarization purposes. The next morning (ZT = 0.5–1.5 h), the standard rodent chow was removed from the food racks. Starting thirty minutes later, the empty dish, an open dish with a HFD pellet, or a closed perforated dish with HFD inside were placed on the bedding inside each cage. A time interval of 5 min was adhered to between each cage with respect to food or dish introduction to accommodate the perfusion-fixation schedule. Time spent interacting with the dishes or with the food was also recorded. The empty dishes and accessible HFD were offered for exactly 30 min, after which the left-over food and dishes were removed from the cage. The dishes with HFD enclosed inside with perforated lids were removed 20 min after placement to prevent the exploration time from exceeding the time usually spent on feeding on HFD (based on prior observations). Each pellet of accessible HFD was weighed before and after the feeding episode and the difference was calculated to determine the amount of food consumed. The mice were then left undisturbed in their cages. At 90 min after the time the HFD and/or dishes were introduced, each mouse received an intraperitoneal injection of euthanasia solution prior to perfusion fixation.

### SACRIFICE AND BRAIN TISSUE PROCESSING

Mice were anesthetized with euthanasia solution (0.1 ml) i.p. and underwent trans-cardiac perfusion with buffered saline followed by fixative solution (4% paraformaldehyde in 0.1 M phosphate buffer, pH 7.4, containing 15% saturated picric acid). Each mouse was perfused with 50 ml fixative for 5 min. Following perfusion, brains were dissected and immersed in the same fixative solution overnight, after which they were transferred to 0.1 M phosphate buffer. Brains were blocked coronally into three equally thick parts using a mouse brain mold. The brain parts were blot-dried and arranged into standard cryomolds (Tissue Tek, #4557) with forebrain parts from four to six different mice encompassing the entire frontal cortex placed in each cryomold. The molds were then filled with warmed 10% gelatin solution, allowed to cool to solidify, and immersed into refrigerated 4% paraformaldehyde solution overnight. The blocks were then removed from the molds, trimmed, glued on polystyrene dishes, and cut into coronal sections (40 μm thick). The sections were collected serially in six-well tissue culture plates such that each well contained a representative series with every 6th section present (distance between adjacent sections in each well is thus 240 μm). Sections were stored at 4°C in 0.1 M phosphate buffer containing 0.1% sodium azide as a preservative prior to the immunohistochemical procedures.

### IMMUNOHISTOCHEMISTRY

One out of six series of sections was stained for Fos immunoreactivity (ir) *using peroxidase staining as well as immunofluorescence procedures* (Gaykema et al., 2007; Gaykema and Goehler, 2009). Phosphate-buffered saline (PBS) was used for all rinses, whereas all antibody solutions were made in PBS containing 0.5% Triton X-100, 0.1% sodium azide, and 1% normal goat serum. First, sections were pretreated with sodium borohydride (0.1%) in PBS for 20 min followed by immersion into 0.3% hydrogen peroxide and 0.1% sodium azide in PBS (30 min) to quench endogenous peroxidase activity. Next, the sections were immersed in blocking solution containing 2% normal goat serum and Fab’ fragment of goat anti-mouse IgG (1:1000) for 4 h at room temperature. Thereafter, interspersed with triple washes in PBS, sections were incubated in anti-Fos (Ab5, EMD Millipore, #PC38, 1:50,000) for 72 h followed by overnight incubation in biotinylated goat anti-rabbit IgG (Jackson ImmunoResearch, 1:1000) with antibodies diluted in PBS containing 0.5% Triton X-100 and 0.1% sodium azide. Subsequently the sections were immersed in avidin–biotin–peroxidase complex diluted in PBS with 0.1% Triton X-100 (ABC Elite kit, Vector; 1:1000, 4 h). Staining was completed using nickel-enhanced 3,3′-diaminobenzidine (DAB, 0.02%, nickelous ammonium sulfate 0.15%) in Tris–HCl (0.05 M, pH 7.6) yielding a black reaction product. Additional sets of sections were stained for Fos ir as described above, followed by staining for one of the following phenotype markers in each set: PV, SOM, and VIP. Sections were incubated in either mouse anti-PV (Sigma, clone PARV-19; 1:5000), rabbit anti-VIP polyclonal antibody (Immunostar #20077; 1:2000), or rat anti-SOM (EMD Millipore #MAB354; 1:2000) for two nights, followed by overnight incubation in either biotinylated goat anti-mouse, anti-rabbit, or anti-rat IgG (Jackson ImmunoResearch; 1:1000), and subsequent incubation in ABC (Vector, 1:1000), and finally reacted with DAB (0.04%) in Tris–HCl buffer yielding brown cytoplasmic staining. Sections from each well were then mounted in sequential order, air-dried, dehydrated through a series of graded ethanol concentrations, cleared in xylene, and coverslipped in DPX.

Another series of sections was used for dual labeling of Fos and special AT-rich sequence binding protein 2 (SATB2), the latter a marker for excitatory neurons in the cerebral cortex (Britanova et al., 2008; Huang et al., 2013). Since both markers are localized in the cell nuclei, determining colocalization required immunofluorescence detection of both epitopes. This set was incubated in a mixture of rabbit anti-Fos (as above) and mouse anti-SATB2 (Abcam, 1:200) for 2 days/nights followed by Cy2-conjugated goat anti-mouse IgG and Cy3-conjugated goat anti-rabbit IgG (Jackson ImmunoResearch; 1:1000) overnight in a light-protected container. Sections from each were then mounted in sequential order, air-dried, dehydrated, cleared, and coverslipped in DPX. During the entire process the slides were protected from light exposure.

Immunohistochemical controls were done on spare sets of sections by omitting one of the primary antisera or replacement by a similarly diluted normal serum, which consistently resulted in absence of immunostaining characteristic for the primary antibodies involved. All antisera were extensively characterized by the manufacturers.

### MICROSCOPIC ANALYSIS

The sections were examined with an Olympus BX51 microscope using 10, 20, and 40× objectives and digital images were captured using a Magnafire digital camera (Optronics, Goleta, CA, USA) and stored images in TIFF format (G4 Apple PowerMac) or loaded into NIH Image (version 1.61) for counting cell nuclei stained for Fos ir. Digital images were cropped and minimally adjusted for brightness and contrast in Adobe Photoshop (version 9.0 in CS2, Adobe Systems, Mountain View, CA, USA).

#### Quantitative analysis of Fos immunoreactivity

In the series stained for only Fos ir, numbers of stained cell nuclei in the mPFC were counted using NIH Image. The mPFC area analyzed corresponded closely with the coronal diagram at 1.9 mm anterior to bregma, just rostral to where the forceps minor of the corpus callosum emerges (Paxinos and Franklin, 2004), and was divided into a dorsal half corresponding with most of the PL and adjacent anterior cingulate cortex (AC) and a ventral half roughly corresponding with the IL and the more ventrally situated dorsal peduncular cortex (DP). Captured using the 20× objective, the digital images covered areas of 425 × 345 μm (height & width), and a 4 × 2 grid of 8 images (2 side-by-side columns of 4 images) each covered the entire span of the mPFC. Both dorsal (PL/AC) and ventral (IL/DP) portions were then covered by 4 images (in a 2 × 2 grid) spanning an area measuring 850 μm × 690 μm. These portions were further subdivided into one column of images covering the superficial layers (L1–3) and another column covering the deep layers (L5–6) of the cortex. Due to the lack of visible landmarks for boundaries, the area and layer subdivisions presented in the results do not precisely follow the boundaries as depicted in the brain atlas, but by using the grid structure instead, care was taken to apply the same sampling in each section and also to avoid double counting. Quantitation was done in NIH Image, where images were equalized in brightness (background subtraction), threshold set for binary conversion, and the number of particles (corresponded to labeled nuclei) counted and recorded. Similarly, Fos expression was assessed in the PVN and Arc nuclei of the hypothalamus. For each, two sections 240 μm apart were chosen with the nuclei prominently present, and the number of Fos-labeled cells were counted bilaterally in each section and summated to yield the total number.

#### Quantitative analysis of double-labeled sets

Using the series of sections with dual peroxidase staining for Fos and either, PV, SOM, or VIP ir, the activation of these subsets of cortical interneurons within the PL/AC and IL/DP was determined by the presence or absence of black nuclear staining for Fos-ir within somata visualized by cytoplasmic staining for PV-, SOM-, or VIP-ir. For this purpose, double-labeled neurons (harboring both brown cytoplasmic and black nuclear reaction products) as well as stained cell bodies lacking the black nuclear staining were counted manually with the use of a 40× objective, and recorded separately for the dorsal (PL/AC) and ventral (IL/DP) halves of the mPFC as well as separately in deep and superficial layers. Stained somata or parts thereof that lacked a discernable nucleus were not included in the counts. Counts from both hemispheres were combined to yield total counts for each portion of the mPFC. For each individual mouse, one section was chosen for analysis that corresponded most closely with the atlas diagram at bregma 1.94 mm (Paxinos and Franklin, 2004).

The series of sections double-labeled for Fos and SATB2 immunofluorescence was evaluated by capturing pairs of images with a 20× objective in a 2 × 4 grid fashion as described above, with the excitation/emission filters alternating between Cy2 and Cy3 fluorophores within each pair. The pairs of images were then exported and stacked in Photoshop, adjusted for brightness and contrast levels, and a third layer was superimposed in which marks were placed overlaying the labeled nuclei that indicate the presence of Fos-ir labeling alone or both Fos and SATB2 labeling within the same nuclei. The markings were counted to yield the number of double-labeled cell nuclei within each cortical subdivision.

### STATISTICAL ANALYSIS

The data time spent inspecting the dish, feeding on HFD, and food-driven exploration (attempting to access the enclosed HFD) were analyzed using ANOVA. Cell numbers and percentages of double-labeled cells were analyzed using ANOVA and Tukey’s post hoc comparisons for each cortical region. In the bar graphs, all grouped values are expressed as means and SE of the mean (SEM). Differences with *p* < 0.05 were considered statistically significant. Statistical analysis and graphs were acquired using Graphpad Prism (v. 6.0e for Mac OS × 10.8).

## RESULTS

### ESTABLISHING THE FOOD INTAKE PARADIGM

**FIGURE 1:**
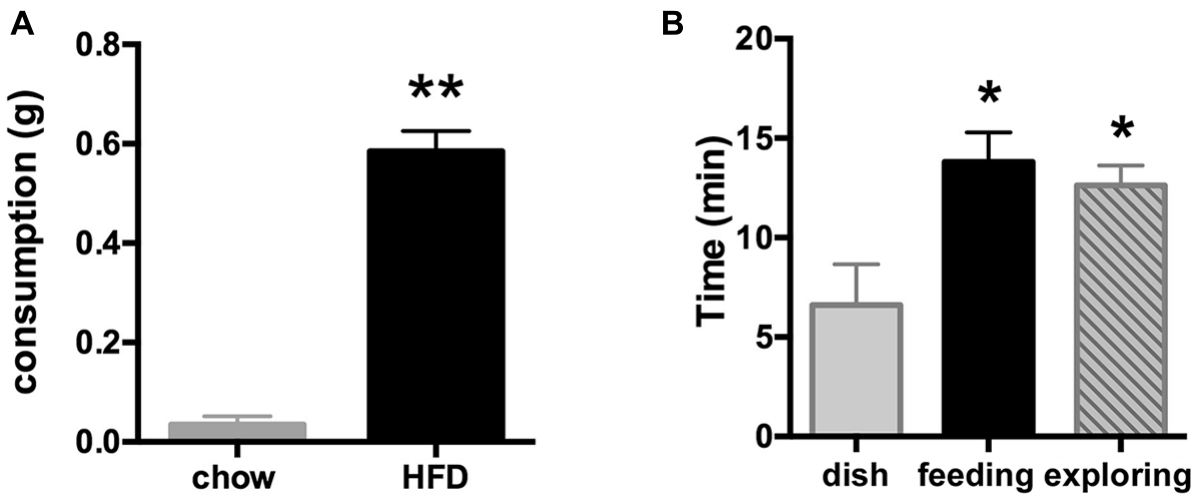
**(A)** Consumption of the palatable HFD (elevated fat and sugar content), but not of regular rodent chow is indicative of a hedonic rather than a metabolic drive. Food was offered shortly after light onset for 30 min starting at ZT = 0.5–1.5 h. **p < 0.001 (*n* = 6/group). **(B)** Introduction of HFD at ZT = 0.5–1.5 h led to a significant increase in amount of time spent on feeding when offered in an open dish bottom (middle black bar) or on food-driven exploration when introduced inside a closed perforated dish (right bar), whereas mice in the control group spent much less time investigating the empty dish (left bar). *p < 0.05 compared to the control group offered an empty dish bottom (*n* = 5 per group).

To ensure adequate food intake and thus Fos expression, we wanted to produce high levels of feeding in the absence of fasting. As the mPFC has been shown to drive food intake in the sated animal, we established an assay using palatable food to stimulate feeding. With the first group of mice, we examined the drive to eat a high fat/high sucrose diet (HFD) that was offered between 30 and 90 min into the light period (between ZT 0.5 and 1.5 h) and compared the amount eaten to the consumption of standard rodent chow by a second group of animals. Indeed, mice offered standard chow consumed none (three out of six) or minimal amounts (three out of six; group average 0.04 ± 0.02 g, 0.11 ± 0.06 kcal), while all six mice offered the highly palatable HFD consumed a significant amount [0.59 ± 0.05 g (3.25 ± 0.28 kcal; **Figure 1A**, *p* < 0.0001]. Thus, for all subsequent studies, the HFD protocol was employed to ensure significant food intake.

### FEEDING- AND FOOD-DRIVEN EXPLORATORY BEHAVIOR-ASSOCIATED FOS EXPRESSION IN THE mPFC

During the food intake assay, the time spent actively feeding on the HFD pellet including minor interaction with the dish bottom (13.8 ± 1.5 min; consumption 0.81 ± 0.09 g) was similar to time spent pursuing the HFD locked inside the perforated dish in the exploratory behavior test (12.6 ± 1.0 min). Time spent eating the accessible or investigating the unobtainable food was significantly greater than time spent engaged with the empty dish bottom in the control group (6.6 ± 2.2 min; ANOVA analysis of group differences: *F*(2,12) = 6.03, *p* = 0.015; **Figure 1B**).

**FIGURE 2:**
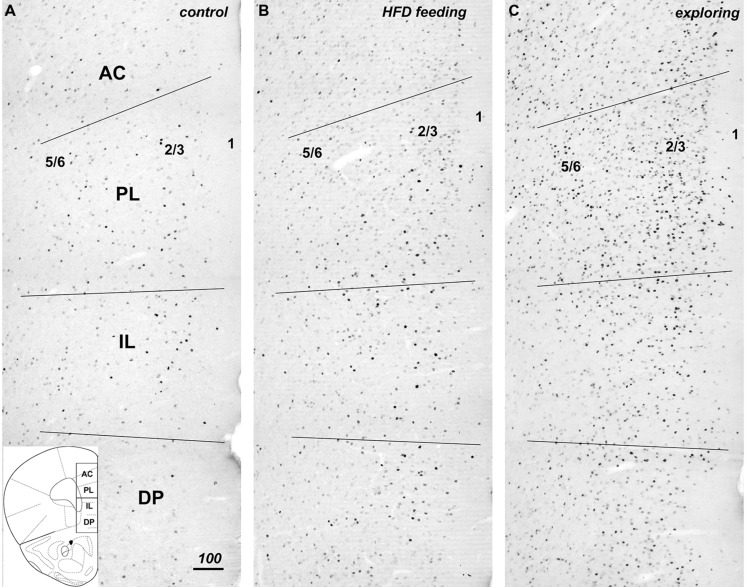
**Compared to controls (A), hedonic feeding on HFD (B) and, to a greater degree, food-driven exploration (C) increased Fos immunreactivity throughout the medial prefrontal cortex from dorsal to ventral to include the anterior cingulate (AC), prelimbic (PL), infralimbic (IL), and dorsal peduncular (DP) subdivisions.** The numbers 5/6, 2/3, and 1 approximate the cortical layers within the photomicrographs taken from the left hemisphere mPFC. The increase in Fos immunreactivity was evident in both deep and superficial layers of these regions **(B,C)**. The coronal diagram in the lower left depicts the corresponding area and includes the dividing line for purpose of analysis of the dorsal and ventral halves. Scale bar in **A** = 100 μm, and applies to all panels.

Introduction of appetitive stimuli that induced either strong feeding or food-driven exploratory behaviors also strongly increased the level of Fos expression in the mPFC (**Figure 2**), with the largest increase in Fos staining seen in the mice that pursued inaccessible HFD (**Figure 2C**). The increase in Fos expression was discernable in all portions of the mPFC, i.e., the dorsal PL/AC as well as ventral IL/DP cortices and extended throughout the superficial (L1–3) and deep layers (L5–6). Within the PL/AC, the Fos-labeled cell nuclei were notably concentrated within the superficial layers 2 and 3, whereas within the IL/DP, a more scattered distribution was observed. This scattered distribution was also seen in both the PL/AC and IL/DP deep cortical layers.

Low to moderate levels of Fos expression were present in all sectors of the mPFC in the control group. Phenotypic characterization and quantification of the cells with Fos-labeled nuclei in both treatment groups revealed a substantial increase in expression in both excitatory neurons and in a subset of inhibitory neurons when compared to control as described below.

### Fos IN EXCITATORY PRINCIPAL EXCITATORY NEURONS (SATB2)

**FIGURE 3:**
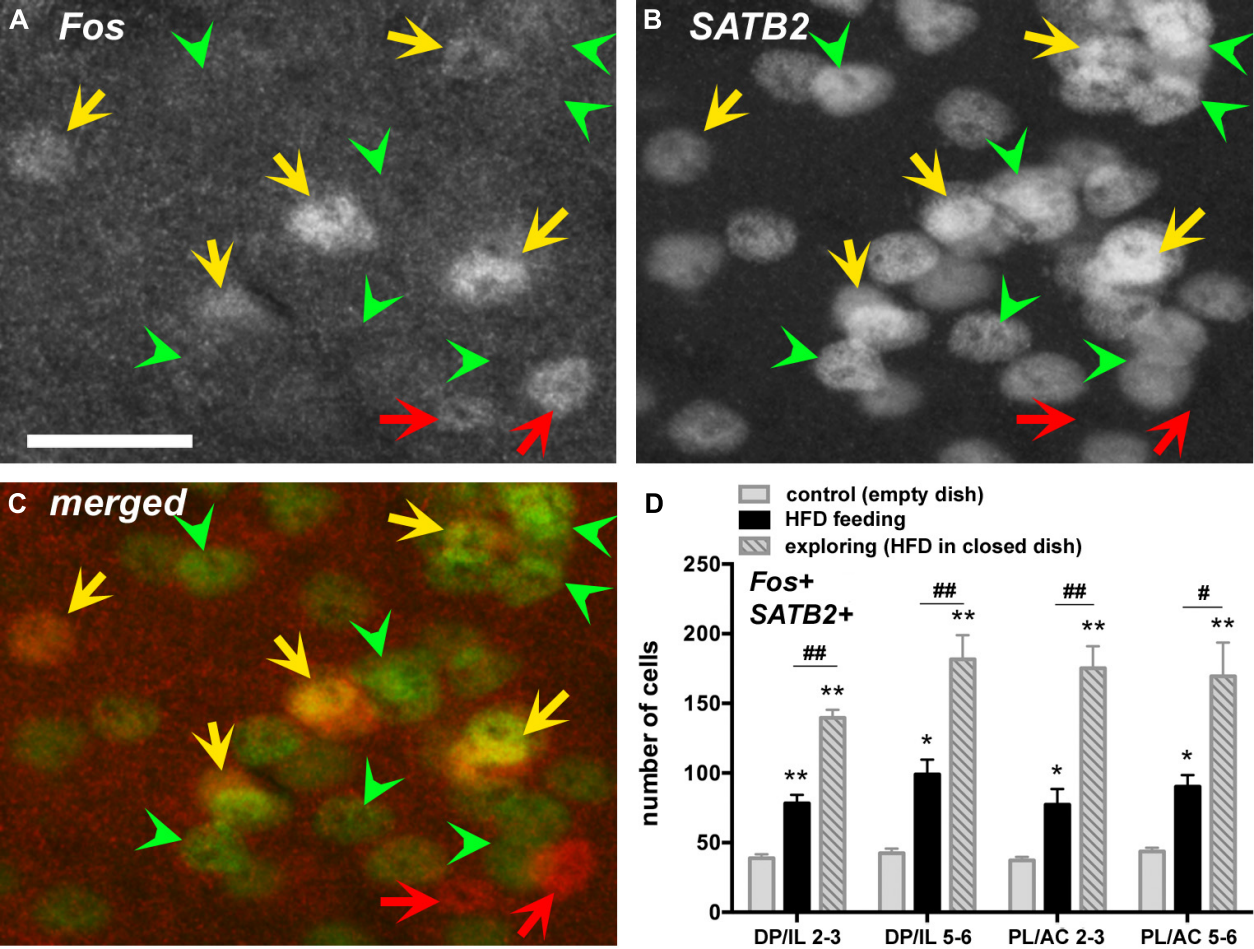
**Many Fos-labeled cell nuclei co-express the nuclear protein special AT-rich sequence-binding protein 2 (SATB2), a marker expressed in principal excitatory neurons but not inhibitory neurons.** Photomicrographs show fluorescent labeling for Fos-ir (in **A**, with Cy3) and for SATB2-ir (in **B**, with Cy2, as well as merged channels in **C**) in layers 2–3 of the PL. Double-labeled cells are indicated with yellow arrows, whereas those only labeled for Fos (and thus negative for SATB2) are indicated with red arrows (putative inhibitory neurons). Some of the SATB2-labeled cells lacking c-Fos labeling are indicated with green arrowheads. **(D)** The number of double-labeled cells (SATB2-positive neurons that show Fos labeling) increased throughout the mPFC in the HFD feeding group, and even more so in the exploratory group in pursuit of the HFD (*n* = 5/group). *p < 0.05, **p <0.005, differences with respect to the control group. ^#^*p* < 0.05, ^##^*p* < 0.005, differences between feeding and exploring groups. Scale bar in **A** = 25 μm, and also applies to **B,C**.

Colocalization of Fos expression with the marker for cortical excitatory neurons SATB2 by immunofluorescence detection revealed that the greater majority (between 80 and 95%) of Fos-positive cells co-expressed SATB2 (**Figures 3A–C**). The number of Fos and SATB2 double-labeled cell nuclei was lowest in the control group, roughly doubled in the HFD feeding group and almost quadrupled in the food-driven exploratory group (**Figure 3D**). These changes occurred uniformly in both the dorsal PL/AC and the ventral IL/DP as well as in the deep (L5,6) and superficial (L2,3) layers.

### Fos EXPRESSION IN DISTINCT INHIBITORY NEURON POPULATIONS (PV, SOM, VIP)

**FIGURE 4:**
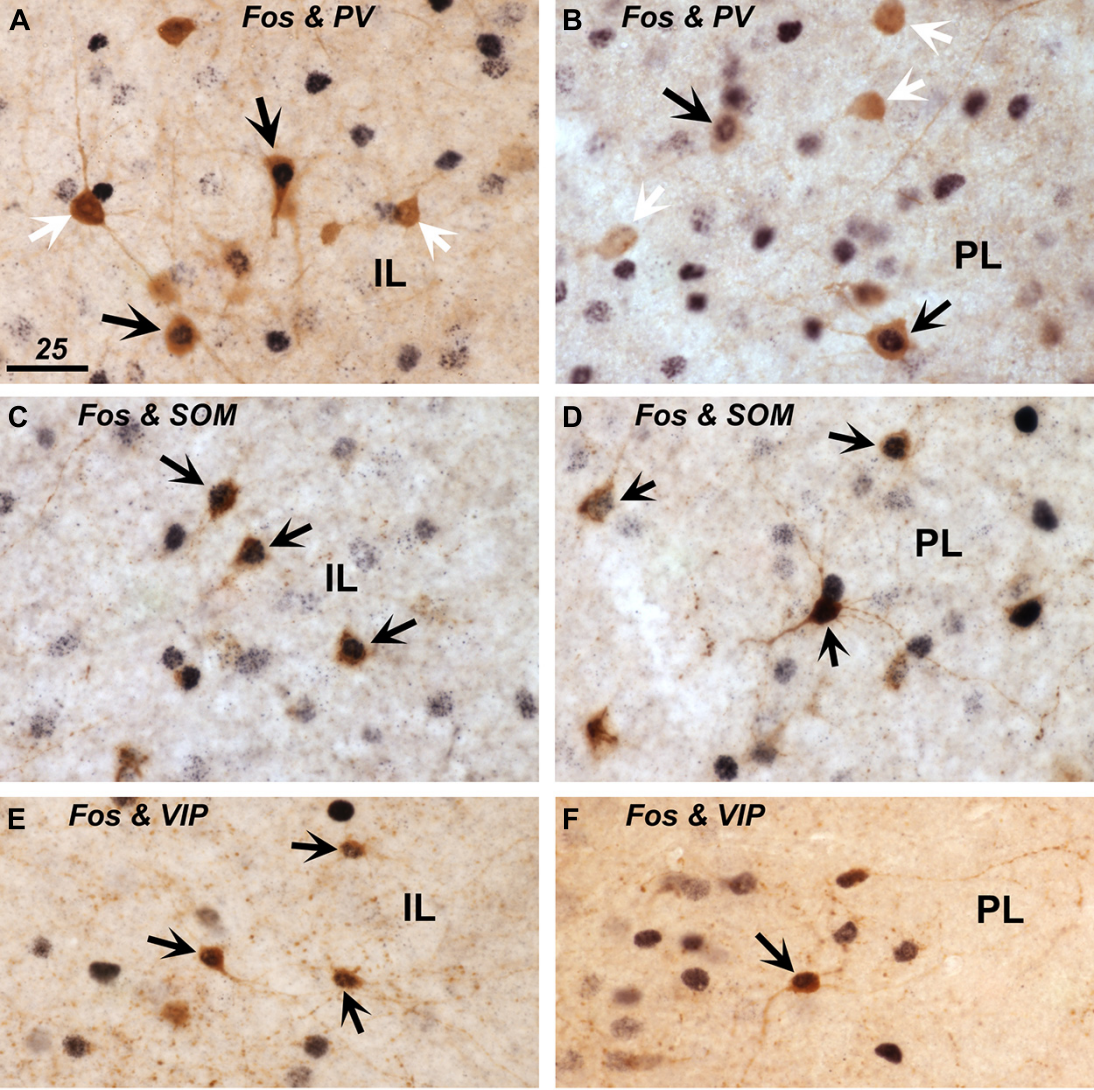
**Expression of Fos protein in inhibitory interneurons immunostained for parvalbumin (PV; in A,B), somatostatin (SOM; in C,D), and vasoactive intestinal polypeptide (VIP; in E,F) within the infralimbic (IL; in A,C,E) and prelimbic cortex (PL; in B,D,F).** Double-labeled cells that show brown cytoplasmic stain and a black nucleus are indicated with black arrows. Single-labeled PV+ cells that lack a black nuclear staining are indicated with white arrows **(A,B)**. Scale bar in **A** = 25 μm, and applies to all panels.

To address the changes in activity of specific populations of inhibitory interneurons in the experimental groups as compared to the controls, we assessed Fos expression in three, non-overlapping groups of interneurons phenotypically identified by the presence of PV, SOM, and VIP ir (shown in representative images in **Figure 4**), which together define the greater majority of all cortical interneurons (Xu et al., 2010). We present both actual counts of double labeled cells as well as percentages of double-labeled cells relative to the total number of interneuron subtype counted (including those that displayed unstained cell nuclei). The latter corrects for variations in total counts in a few instances between the experimental groups, but in general both methods of comparisons yielded similar differences between the experimental groups. However, presentation of data in the form of percentages of double-labeled cells provide further insight as to how prominently certain subsets of interneurons are engaged, while this also corrects for uneven distribution of the interneuron subtypes across the cortical layers. Whereas the SOM-ir cells were distributed rather evenly across areas and layers, PV-ir neurons were predominantly present in the deep layers 5–6, and the VIP-ir cells were mostly encountered in the superficial layers 1–3.

#### Parvalbumin

**FIGURE 5:**
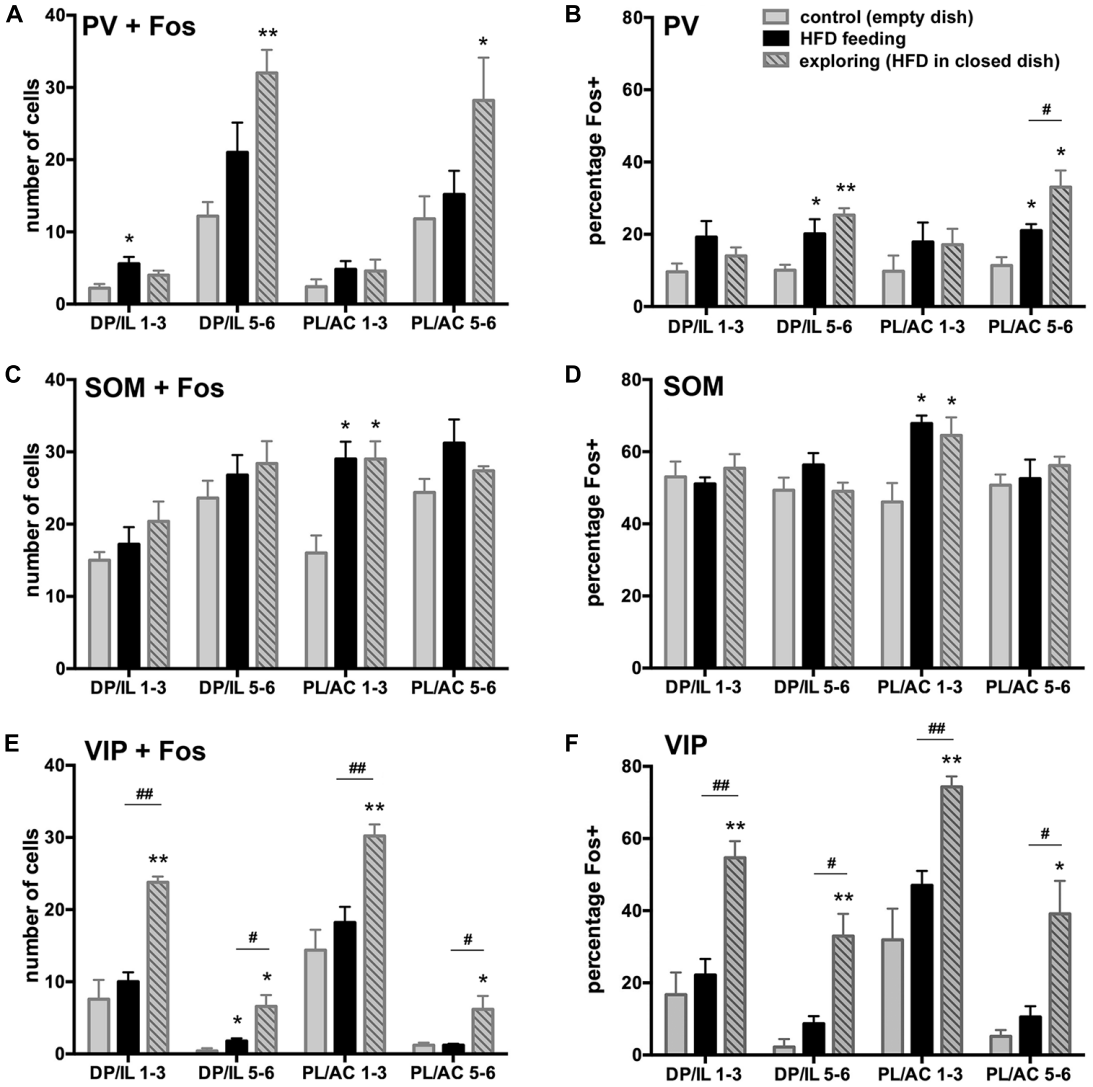
**Fos expression in inhibitory interneurons and differences among the experimental groups vary among the three major non-overlapping subpopulations in the medial PFC (PV in A,B, SOM in C,D, VIP in E,F). A,C,E** depict the number of double-labeled cells counted, whereas **B,D,F** show the percentage of the interneuron subpopulations that showed Fos-ir. **A,B**: HFD exposure led to an increase in Fos-ir in PV neurons most of which were in the deep layers of the DP/IL and PL/AC, and this increase was robust only in the food-driven exploration group. **C,D**: SOM-labeled neurons in layers 1–3 of the PL/AC showed comparable increases in Fos-ir among the HFD feeding and exploring groups, but no differences between groups were apparent in the other parts of the mPFC, where about half of all SOM-labeled cells were Fos-positive. **E,F**: Among the VIP neurons, a robust increase in Fos-ir occurred in the food-driven exploration group, whereas HFD feeding led to a small but insignificant rise. *p < 0.05, **p < 0.005, differences with respect to the control group;^#^*p* < 0.05, ^##^*p* < 0.005, differences between feeding and exploring groups.

A small portion of PV-ir neurons exhibited Fos-ir in the control group, and a modest increase in Fos expression among the HFD feeding and food-driven exploratory groups occurred in the deep layers of the mPFC, which harbor the majority (75–80%) of PV-labeled cells (**Figures 5A,B**). This increase was significant both in terms of numbers of double-labeled cells and percentage of all PV+ cells (DP/IL L5-6: cell counts: *F*(2,12) = 9.48, *p* = 0.003; percentage: *F*(2,12) = 8.19, *p* = 0.006; PL/AC L5-6: cell counts: *F*(2,12) = 4.04, *p* = 0.046; percentage: *F*(2,12) = 12.04, *p* = 0.001). The numbers and percentages were not significantly different in the superficial layers where only ∼20% of PV+ cells are located. HFD feeding and food driven exploration both increased the percentage of PV cells that were Fos-positive from approximately 10% in the controls to 20% in the feeding group (*p* < 0.05), and more robustly in the food driven exploration group to 25% and 33% in the deep layers of the DP/IL and the PL/AC, respectively (*p* < 0.005).

#### Somatostatin

SOM-ir interneurons were fairly uniformly distributed across the different cortical layers of both the ventral and dorsal portions of the mPFC and as a result so were the SOM and Fos double-labeled cells (**Figure 5C**). A large proportion (about half) of the SOM-ir cells were Fos-positive throughout all subdivisions in all experimental groups (**Figure 5D**). The only difference between the experimental groups was encountered in the superficial layers 1–3 of the PL/AC, where both HFD feeding and food-driven exploration led to a further increase in the number of double-labeled cells [*F*(2,12) = 9.49, *p* = 0.003] as well the percentage of all SOM+ cells that were Fos-positive [*F*(2,12) = 7.21, *p* = 0.009]. Here, the percentage of SOM+ cells that were Fos-positive increased from 46 ± 5% in controls to 68 ± 2% in the HFD feeding group (*p* < 0.05) and to 65 ± 5% in the food driven exploration group (*p* < 0.05).

#### Vasoactive intestinal peptide

A relatively small proportion of VIP-ir interneurons, most of which are located in the superficial layers 1–3, exhibited Fos-ir in the control group, whereas a significant increase in the numbers and percentages of VIP and Fos double-labeled cells was seen in the food-driven exploratory group, but little increase was seen in the HFD feeding group (**Figures 5E,F**). This increase in the exploratory group was significant both in terms of numbers of double-labeled cells and percentages of VIP/Fos+ cells (DP/IL L1–3: cell counts: *F*(2,12) = 19.4, *p* = 0.0002; percentage: *F*(2,12) = 16.0, *p* = 0.0004; PL/AC L1–3: cell counts: *F*(2,12) = 13.4, *p* = 0.0009; percentage: *F*(2,12) = 14.0, *p* = 0.0007). In the control group, the percentage of VIP-labeled cells that exhibited Fos-ir in layers 1–3 ranged from 17 ± 6% in the DP/IL to 32 ± 8% in the PL/AC. Food driven exploratory activity led to a large increase in Fos-ir, with percentages of 55 ± 5 in the DP/IL (*p* = 0.001) and 74 ± 3% in the PL/AC (*p* = 0.002). Although the numbers of VIP and Fos double-labeled cells were small in the deeper layers of the mPFC, the food driven exploratory activity led to a significant increase in the proportion of double-labeled cells here as well [to 32% in the DP/IL layers 5–6: *F*(2,12) = 16.7, *p* = 0.0003; to 39% in the PL/AC layers 5–6: *F*(2,12) = 10.4, *p* = 0.002].

### Fos EXPRESSION IN THE PARAVENTRICULAR AND ARCUATE NUCLEI OF THE HYPOTHALAMUS

**FIGURE 6:**
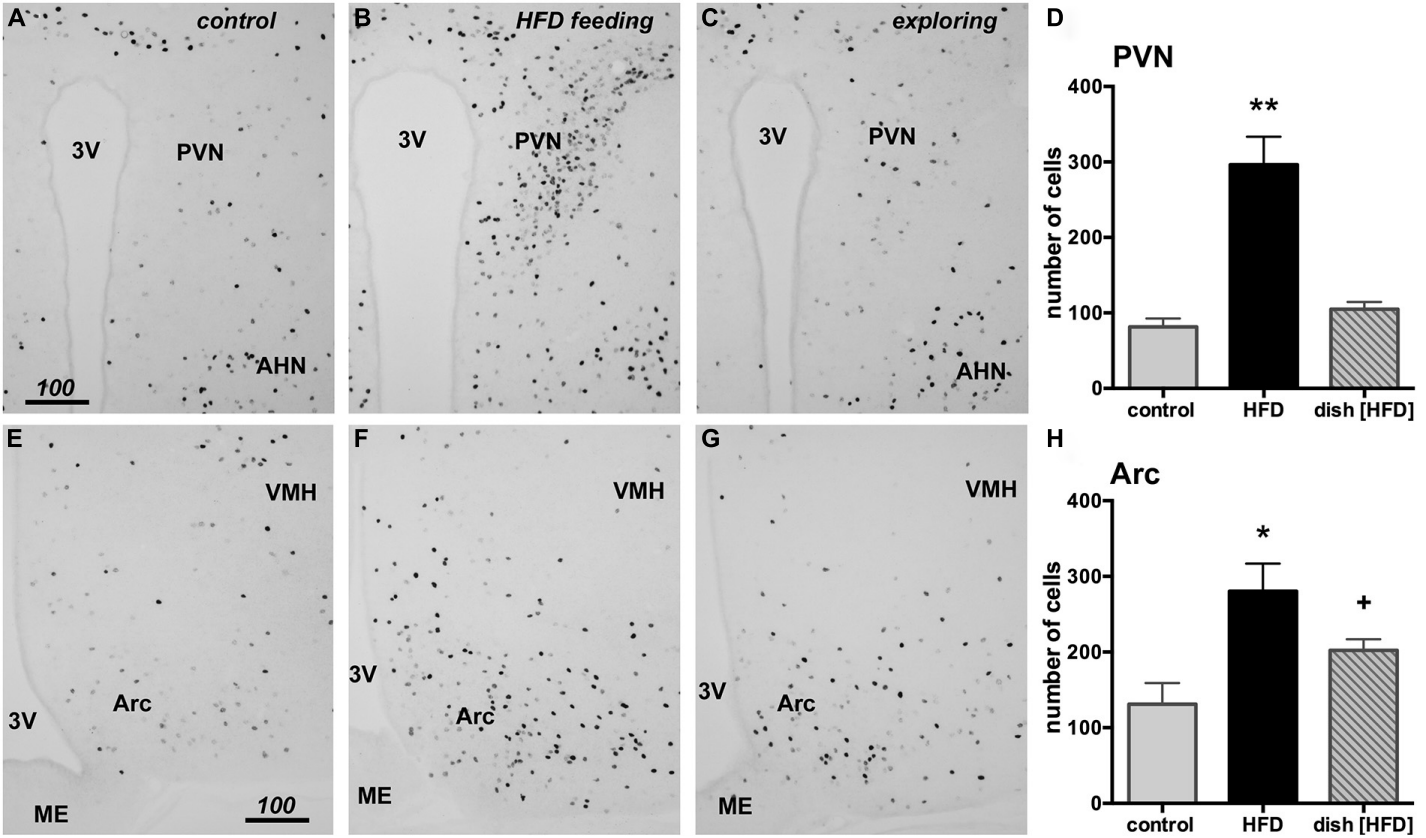
**HFD consumption, but not food-driven exploration, substantially increases Fos in the paraventricular nucleus (PVN) and the arcuate nucleus (Arc) of the hypothalamus when compared to the control group (exposed to empty petri dish).** Representative photomicrographs are shown of the PVN **(A–C)** and the Arc **(E–G)** from control **(A,E)**, HFD fed **(B,F)** and animals following food driven exploratory behavior **(C,G)**. HFD feeding led to a significant increase in Fos in the PVN (**D**, **p < 0.05) and the Arc (**H**, *p < 0.05). No change in fos expression was observed in the PVN of the food driven exploratory behavior group (**D**, dish [HFD]) while a small, yet statistically insignificant increase was noticeable in the Arc (**H**, +). Scale bars in **A,E** = 100 μm and also applies to panels **B,C** and **F,G**.

The hypothalamic PVN and Arc nuclei have both been implicated in the control of food intake. To examine whether HFD intake and food driven exploratory activity differentially activated neurons within these brain areas, we performed counts of Fos positive neurons using tissue taken from the same brains used in the prefrontal cortical analyses. Although a significant increase in expression was observed in the HFD group relative to control [*F*(2,12) = 26.25, *p* < 0.0001], no increase in PVN fos expression was seen in mice following food driven exploration (**Figure 6**). This same result was also observed in the Arc, where HFD intake increased Fos expression relative to control [*F*(2,12) = 7.175, *p* = 0.0089]. Food driven exploratory activity, meanwhile, only modestly increased Fos expression in the Arc that did not reach statistical significance (+; **Figure 6**).

## DISCUSSION

The present investigation demonstrates that ingestion of highly palatable food and behavior directed at exploring food reward differentially engages areas and cell types of the mPFC. Although our analysis was limited to an investigation of the induction of Fos protein as a read-out for neuronal activation and thus does not permit an assessment of acute changes in neuron activity, it demonstrates important differences in how stimuli can modulate PFC function. Specifically, while an increase in Fos expression was observed in the PL/AC and IL/DP in both behavioral paradigms relative to the control condition, the magnitude of Fos induction (in terms of cell number) and the contribution by the different cell types across these subdivisions and layers of the mPFC showed significant variation.

Excitatory pyramidal neurons throughout the PL/AC and IL/DP, as identified with SATB2 ir, showed a significant increase in Fos expression across all cortical cell layers 2–6. Interestingly, food dependent exploratory behavior produced a significantly greater increase in Fos expression among the SATB2-positive neurons when compared to either palatable food intake or the control condition. This difference could be explained by the increased attentional and cognitive processing engaged during the food-exploratory task that differentially activates the PFC when compared to the intake of freely available food. As hypothesized in a prior study, the mPFC is more likely to show greater entropy or complexity in activity when considering multiple strategies for obtaining reward during food seeking or exploration than during the consumption of easily available food when only one outcome has to be considered (Caracheo et al., 2013).

Most likely, this pattern of enhanced Fos expression in pyramidal neurons during the exploratory task and food intake will also emerge following the execution of other tasks that require varying degrees of mPFC activation. Clearly, the question of whether the increased number of neurons recruited during the exploratory task is required to produce specific increases in behavior output and whether these particular changes in Fos also occur during non-appetitive behaviors requires further investigation. For example, it would also be important to investigate whether these same neurons are engaged to reduce behavioral responding, as observed during eyeblink conditioning (Leal-Campanario et al., 2007) and during tests of impulsive behavior that activate select regions of the mPFC (Chudasama et al., 2003; Kim and Lee, 2011). One would expect that this would be the case, i.e., activation of mPFC neurons would differentially affect behavioral responding, capable of producing both enhancements and decrements in select behaviors that ultimately maximize correct goal selection. In particular, this would be expected to be the case for neurons residing in the IL/DP that are involved in the suppression of behavioral responding [mostly in the case of maladaptive or inappropriate behaviors that prevent goal attainment (Quirk et al., 2000; Peters et al., 2008; LaLumiere et al., 2010; Smith and Graybiel, 2013)]. The PL/AC cortex, meanwhile, is often thought to drive behaviors that enhance goal attainment (McLaughlin and See, 2003; Naneix et al., 2009).

It also remains to be determined whether principal excitatory mPFC neurons that project to specific target sites are differentially activated following specific behaviors. For example, subsets of mPFC excitatory neurons that project to, e.g., the striatum, basolateral amygdala, hypothalamus and midline/intralaminar thalamus would be expected to be engaged by the exploration, pursuit, and ingestion of palatable food (Ongur and Price, 2000; Gabbott et al., 2005) and contribute to the rise in Fos expression. However, as the increases in pyramidal neuron Fos expression were evenly distributed throughout the mPFC, it is difficult to predict whether this differential activation occurs. For example, prior work has shown that projections from the rat mPFC to the striatum are organized in a dorsal to ventral pattern, with the PL/AC preferentially innervating the dorsal striatum and the IL/PD showing greater innervation of the ventral striatum (Gabbott et al., 2005). Innervation of the basolateral amygdala, meanwhile, originates from pyramidal neurons residing in superficial cortical layers 2–3 of the IL/PD and in the more superficial areas of layer 5 in the PL/AC (Gabbott et al., 2005). Since we did not see any obvious difference in regional Fos expression in either the HFD or food driven exploratory conditions, we cannot speculate whether selective neuronal activation is occurring in the subcortical nuclei shown to modulate behavior, in the absence of conducting immonohistochemical experiments in combination with retrograde tracing.

Interestingly, unlike the excitatory neurons of the mPFC, inhibitory interneuron Fos expression did not occur uniformly across the prefrontal cortex. Among the three chemically distinct and non-overlapping populations of inhibitory interneurons characterized by the expression of PV, VIP and SOM, the increased magnitude of Fos expression did not recapitulate that observed in the excitatory neurons, as we did not observe uniform changes throughout the mPFC of the mice engaged in food-driven exploratory activity. Furthermore, differences in Fos expression between treatment groups varied widely with each of the three subpopulations of interneuron. While expression of Fos was marginally increased in PV-labeled interneurons following food intake, larger increases were seen following food driven exploration, and mainly involved the PV cells in the deep layers 5–6 of the mPFC. Similarly, Fos expression in VIP neurons showed little change following food intake whereas the food-driven exploratory activity produced a significant increase in Fos in VIP cells in all regions and layers examined. In contrast, expression of Fos in SOM neurons showed little change with either treatment, except for a modest increase in Fos expression in SOM neurons in superficial layers of the PL/AC. It should be noted that SOM neurons expressed Fos at much higher levels in all behavioral contexts throughout the mPFC, including the control group, which may reflect an active inhibitory role for this subset of interneurons during quiet wakefulness (Gentet et al., 2012).

How might differential activation of inhibitory neurons shape mPFC pyramidal neuron excitability and neuronal output? A portion of PV neurons receive extensive innervation from both inhibitory interneurons, among them VIP (Pi et al., 2013) and SOM cells (Pfeffer et al., 2013), and from excitatory pyramidal neurons, acting to initiate the rhythmic discharge of pyramidal cells (Sohal et al., 2009) as pyramidal neuronal pacemakers (Somogyi et al., 1983). The induction of Fos in PV neurons therefore likely follows significant pyramidal neuron activation during food seeking. However, the fact that a lesser magnitude of pyramidal neuron activation associated with feeding on HFD produced a minimal increase in Fos in PV neurons was interesting, as it suggests that the degree of pyramidal neuron engagement determines a threshold for PV neuronal transcriptional activation of the c-*fos* gene. Furthermore, it remains to be determined how Fos and other transcriptional changes in the PV neuron affect the ability of this cell type to enhance cortical information processing (Cardin et al., 2009).

VIP-containing interneurons, meanwhile, comprise primarily radial cells in superficial layers extending their axons between mPFC cortical layers (along with a small population of small basket-like cells; Kawaguchi and Kubota, 1996), coordinating the activity of neurons within cortical columns (Mountcastle, 1997) acting to enhance pyramidal neuron activity (Pi et al., 2013). Similarly to PV neuron activation, VIP cells showed little or no enhanced Fos induction in the feeding group despite significantly activated pyramidal mPFC neurons, suggesting that high levels of VIP neuron activation may not be required for mPFC modulation of feeding behavior, or that the magnitude of increase in activity did not reach the threshold to initiate c-*fos* transcription. We can also infer that the effect of VIP on food intake in the mPFC likely differs from the effects of this peptide in other areas of the brain, as intracerebroventricular injection of VIP in chicks has been shown to result in decreased feeding (Khan et al., 2013).

Unlike PV neurons, VIP interneurons receive significant inputs from superficial cortical neurons and from projections originating outside of the mPFC (Arroyo et al., 2012) while also receiving innervation from pyramidal neurons within the cortical column similar to that seen in somatosensory cortex (Lee et al., 2013). VIP neuron activation would then be expected to enhance pyramidal neuron activity through a recently described disinhibitory mechanism as they target other inhibitory interneurons including those expressing PV (Pi et al., 2013). Interestingly, the disinhibitory action of VIP neurons would then be expected to reduce Fos expression among PV neurons, something that we didn’t observe in the mice engaged in food-driven exploratory behavior. Our observations raise the possibility that increases in Fos expression in the two populations occurred with different time courses, indistinguishable using the Fos immunohistochemistry approach that lacks the necessary temporal resolution. VIP neuron activation may initially inhibit PV neurons that may in turn receive excitatory feedback from the glutamatergic pyramidal cells, a hypothesis that must be tested in future experiments. However, it is also possible that VIP interneurons only target a subset of PV+ cells (as reported by David et al., 2007) and preferentially those in the superficial layers 2 and 3 limiting the VIP cells’ disinhibitory control to those superficial PV cells (Letzkus et al., 2011). It remains to be determined whether organizational microcircuitry principles thus far determined for areas such as the auditory and visual sensory cortices in the mouse also generally apply to the mPFC, as cortical column organization could differ substantially between different cortical areas. Finally, with respect to both the VIP and PV neuronal populations, future experiments will also be required to test how activation of these interneurons is necessary for the correct modulation of mPFC-dependent behaviors.

Unlike that observed in the other cell types investigated, less variation was observed between treatments in the SOM-expressing cells. SOM expression is often seen in Martinotti cells that provide dendritic inhibition of pyramidal neurons controlling their input (Fanselow et al., 2008; Kvitsiani et al., 2013).

While functioning in a manner similar to PV neurons, the SOM cells show activation with a significantly different time course, consistently showing an enhancement in theta rhythm and significant coupling with other SOM expressing cells (Fanselow et al., 2008). Acting as a brake on mPFC neuronal activity, SOM interneurons produce inhibition within a narrow dynamic range. As a result, potential differences in the nature and strength of the stimulation that occurs following food driven exploration or ingestion may not translate into differences throughout the mPFC in transcriptional activation of the c-*fos* gene in the SOM interneuron. Interestingly, SOM administration in chicks has been shown to enhance food intake (Tachibana et al., 2009), suggesting that either SOM action in the mPFC may in fact be important in supporting food intake and food-driven exploration or that, more likely, SOM acts at other CNS sites to promote these behaviors.

While HFD intake and food-driven exploration produce differing effects on Fos expression in the prefrontal cortex, we also wanted to investigate whether these tasks would selectively enhance Fos expression in the hypothalamus. Interestingly, we observed an increase in PVN and Arc Fos expression following HFD intake and not during food-driven exploration. Our work illustrates that, despite the animal being able to see and smell the HFD, this stimulation alone was not sufficient to induce Fos expression in nuclei shown to regulate food intake. This finding is in agreement with observations that PVN (Mitra et al., 2011) and Arc (Hsu et al., 2010) neurons are not activated in anticipation of palatable food intake. Our observations also reinforce the hypothesis that the food-driven exploration task does not simply produce pan-neuronal activation but selectively modulates neurons involved in driving reward seeking. Furthermore, the lack of Fos induction in the PVN in particular suggests that the animal does not experiencing frustration (Latham and Mason, 2010) or an enhancement in stress (O’Mahony et al., 2010) during the food-driven exploration task.

In conclusion, our work, in concert with the other studies discussed above, highlights the complexities of how different contextual stimuli modulate prefrontal cortical function. While the degree of neuronal activity leading to Fos induction increases uniformly among principal excitatory neurons of the mPFC, interneuron activation showed significant differences in terms of the magnitude of Fos induction. Our observation that increased Fos expression in VIP interneurons, and to a lesser extent in PV neurons, was associated with a heightened elevation in pyramidal neuron activity following food-driven exploratory behavior suggests that transcriptional along with functional activation of these interneurons may mediate this elevation in excitatory neuron activity. Thus, we believe that the induction of states of heightened mPFC neuronal activity associated with high cognitive demand requires the elevated functional and transcriptional participation of PV- and VIP-expressing interneurons.

## Conflict of Interest Statement

The authors declare that the research was conducted in the absence of any commercial or financial relationships that could be construed as a potential conflict of interest.
